# Management of Oral Lichen Planus in a Patient With Comorbidities: A Case Report

**DOI:** 10.7759/cureus.108501

**Published:** 2026-05-08

**Authors:** Syed Fareed Mohsin

**Affiliations:** 1 Department of Oral and Maxillofacial Diagnostic Sciences, Qassim University, Buraidah, SAU

**Keywords:** corticosteroids, desquamative gingivitis, glycemic control, malignant transformation, oral lichen planus, type 2 diabetes mellitus

## Abstract

Oral lichen planus is an inflammatory, immune-mediated mucocutaneous disease characterized by multiple clinical presentations, mainly reticular and erosive types. The association between lichen planus and diabetes mellitus has been widely accepted, which can aggravate the disease severity and affect treatment outcome. A 42-year-old male of Asian descent presents with type 2 diabetes mellitus and hyperlipidemia, having pain and burning sensation, which was exacerbated by hot and spicy foods. Intraoral clinical examination revealed bilateral white striations and erosive lesions on the buccal mucosa, gingiva, and labial vestibule; the clinical diagnosis was consistent with reticular and erosive lichen planus with desquamative gingivitis. The resistance to prior therapy and increased intensity of symptoms were exacerbated by immune dysregulation and persistent inflammation due to type 2 diabetes mellitus. The patient was treated with multiple therapies, including intralesional, topical, and systemic steroids, resulting in relief of symptoms, and significant clinical improvement was observed after six weeks. The management of lichen planus with systemic comorbidities emphasizes the significance of a customized treatment plan and interdisciplinary care. Despite the potential for malignant transformation, it is important to implement long-term follow-up.

## Introduction

Oral lichen planus (OLP) is a chronic immune-mediated disorder that affects the oral mucosa and skin, characterized by remission and exacerbation. It has six different clinical types; however, reticular and erosive are the most common, and lead to pain and discomfort [1].

The precise etiology of OLP is unknown; it is believed to result from a T-cell-mediated autoimmune response that targets the basal layer of the oral epithelium. Contributing factors can be linked to habits like chewing betel nuts, drinking alcohol, and smoking; multiple disease processes, such as viral and bacterial infections, autoimmune disease, and hepatitis C virus infection; and systemic diseases, such as diabetes mellitus. Skin lesions are found in severe cases and might affect the oral and genital regions [2]. 

Diabetes mellitus, especially type 2 diabetes mellitus (T2DM), is a prevalent chronic metabolic disorder and has been associated with an increased prevalence of OLP, as well as with more severe manifestations of this disease. The association between DM and OLP was first reported by Grinspan et al in 1966; subsequently, several studies confirmed a significant relationship between DM and OLP [3]. The findings indicate that the prevalence of OLP in diabetic patients is twice as high as in non-diabetic individuals, suggesting a bidirectional relationship. Poor glycemic control has been linked to more severe and resistant lesions. The association of underlying mechanisms likely involves immune dysregulation and chronic inflammation, both of which are hallmark features of DM [4,5].

The main concern associated with OLP is its potential for malignant transformation. Recent studies explored the molecular mechanisms linking OLP to cancer development. Chronic inflammation and immune dysfunction in OLP may foster an environment favorable to malignant transformation, especially in DM, which aggravates the disease progression [6,7].

Managing OLP in individuals with systemic disease like DM requires a comprehensive strategy. Various treatment modalities for OLP have been used, which range from topical, intralesional, and systemic corticosteroids to more sophisticated therapies such as laser treatment, which suggests that every individual management plan should be tailored to the severity of symptoms. For diabetic patients, attaining optimal glycemic control is essential to reduce the severity and improve overall outcomes [8].

This case report aims to highlight the therapeutic response of refractory OLP in a diabetic patient using combined steroid therapy. It also emphasizes the importance of an interdisciplinary approach to managing patients with comorbidities.

## Case presentation

A 42-year-old male of Asian descent presented to the dental clinics of the College of Dentistry at Ar-Rass, Qassim University, in OCT 2025, with the chief complaint of severe generalized pain and burning sensation while consuming seasoned food, resulting in functional impairment with eating and maintaining oral hygiene. Despite being treated with various medications for about 4-6 weeks, the symptoms persisted. The patient had no history of chewing tobacco, smoking, or alcohol, and was negative for hepatitis C virus (HCV) and hepatitis B virus (HBV). The patient's medical history was significant for type 2 diabetes mellitus (T2DM) for the last 10 years and hypercholesterolemia, for which he was prescribed oral hypoglycemic medications vildagliptin and metformin hydrochloride (HCL), and anti-cholesterol medication rosuvastatin 20 mg. The patient’s past dental history includes extractions, multiple restorations, and root canal therapy.

**Figure 1:**
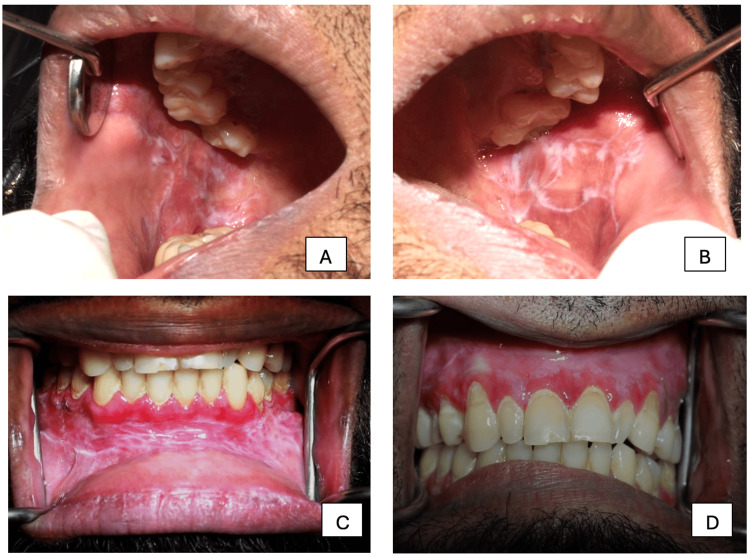
Lesion on right buccal mucosa (A), left buccal mucosa (B), mandibular gingiva (C), and maxillary gingiva (D)

Laboratory investigations revealed that complete blood count parameters were within normal range, glycated hemoglobin (HbA1c) was 8.5%, and fasting blood glucose was 197 mg/dl. The patient was advised to consult an endocrinologist to control the blood glucose level. Extraoral clinical examination showed no evidence of cutaneous lesions or palpable lymphadenopathy. Intraoral examination revealed widespread non-scrapable white striation and erosive lesions involving the right and left buccal mucosa, labial vestibule, and gingiva (Figure 1). Based on the clinical examination by ruling out the differentials of lichenoid reactions, systemic lupus erythematosus, erythema multiform, and autoimmune disorders, the patient was given a provisional diagnosis of reticular and erosive forms of oral lichen planus (OLP), accompanied by desquamative gingivitis.

**Figure 2:**
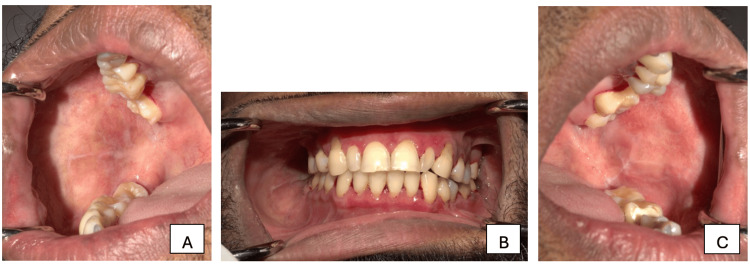
Post-treatment right buccal mucosa (A), anterior view, (B) left buccal mucosa (C)

After explaining the case to the patient and obtaining informed consent, a treatment plan was formulated. Patient received an intralesional injection of dexamethasone sodium phosphate into the buccal mucosa for 6 weeks, along with topical steroid gel (triamcinolone acetonide) applied to the gingiva. In addition, systemic prednisolone 10 mg/day (5 mg twice daily) was administered for three weeks, followed by a tapering dose of 5 mg/day for one week; afterwards, the medication was discontinued. Following the regimen for faster and effective disease control, the lesions showed gradual healing. At the end of six weeks, complete resolution of lesions was observed with no reported pain or burning sensation (Figure 2). The patient was advised to return for follow-up visits at three-month, six-month, and one-year intervals to monitor for recurrence and any dysplastic changes.

## Discussion

Oral lichen planus (OLP) is a chronic inflammatory mucocutaneous condition caused by an autoimmune response mediated by T-cells targeting basal keratinocytes. The present case highlights the clinical challenges of managing reticular/erosive OLP in a patient with systemic comorbidities, including T2DM and hyperlipidemia.

The clinical presentation showed bilateral white non-scrapable striations on the buccal mucosa with erosive lesions, which align with the characteristic features of reticular and erosive types of lichen planus. Erosive lichen planus is associated with considerable discomfort when consuming hot or spicy food [9]. This type exhibits greater resistance to therapy and requires aggressive therapeutic intervention [2].

An important aspect of this case is the presence of T2DM. Recent data indicate a significant association between OLP and diabetes mellitus compared to non-diabetic individuals. Improper glycemic control may worsen the severity and duration of OLP lesions due to persistent inflammation and immunological dysfunction, which elucidates the inadequate response to prior treatment in this case [4].

Intralesional steroids, including dexamethasone, are effective in managing localized lesions, whereas systemic corticosteroids are designated for resistant lesions. In addition, topical triamcinolone was also included, which has a potent anti-inflammatory role in controlling the severity of symptoms. The present therapeutic strategy used in this case aligns with the contemporary guidelines for moderate to severe OLP [8].

The malignant transformation rate of OLP to oral squamous cell carcinoma is another factor to consider. The transformation rate of approximately 1.8% has been reported. It is believed to be influenced by chronic inflammation, epithelial dysregulation, and immune alterations, while studies have emphasized the need for long-term follow-up, particularly in erosive lichen planus [6,7,10].

A limitation of this case report was the lack of histopathological confirmation, as the patient was apprehensive and refused to undergo a biopsy. The diagnosis of OLP was made based on the typical clinical presentation, and management was initiated accordingly.

## Conclusions

Metabolic and inflammatory pathways may influence the risk in patients with systemic conditions such as diabetes mellitus and hyperlipidemia. Consequently, it is essential to implement interdisciplinary management to involve an endocrinologist, which will add substantial value to the OLP management. It is equally important to optimize blood glucose levels, as it influences the treatment outcomes and decreases severity. This case emphasizes the significance of patient education and an individualized treatment plan; however, long-term monitoring is imperative for any dysplastic changes.
